# Cryo-thermal therapy elicits potent anti-tumor immunity by inducing extracellular Hsp70-dependent MDSC differentiation

**DOI:** 10.1038/srep27136

**Published:** 2016-06-03

**Authors:** Jun Zhu, Yan Zhang, Aili Zhang, Kun He, Ping Liu, Lisa X. Xu

**Affiliations:** 1 The School of Biomedical Engineering and Med-X Research Institute, Shanghai Jiao Tong University, Shanghai, China; 2 Neurosurgery Department, Ruijin Hospital,School of Medicine, Shanghai Jiao Tong University, Shanghai, China

## Abstract

Achieving control of metastatic disease is a long-sought goal in cancer therapy. Treatments that encourage a patient’s own immune system are bringing new hopes in reaching such a goal. In clinic, local hyperthermia and cryoablation have been explored to induce anti-tumor immune responses against tumors. We have also developed a novel therapeutic modality of cryo-thermal treatment by alternating liquid nitrogen (LN2) cooling and radio frequency (RF) heating, and better therapeutic effect was achieved in treating metastatic cancer in animal model. In this study, we investigated the mechanism of systemic immune response elicited by cryo-thermal therapy. In the 4T1 murine mammary carcinoma model, we found that local cryo-thermal therapy resulted in a considerable reduction of distant lung metastases, and improved long-term survival. Moreover, results of tumor re-challenge experiments indicated generation of a strong tumor-specific immune memory after the local treatment of primary tumors. Our further study indicated that cryo-thermal therapy caused an elevated extracellular release of Hsp70. Subsequently, Hsp70 induced differentiation of MDSCs into mature DCs, contributing to the relief of MDSCs-mediated immunosuppression and ultimately the activation of strong anti-tumor immune response. Our findings reveal new insight into the mechanism of robust therapeutic effects of cryo-thermal therapy against metastatic cancers.

Metastasis is the major cause of mortality in breast cancer patients. The 5-year survival rate is only 23% after the diagnosis of stage IV breast cancer^1^. While conventional therapeutic modalities such as surgery, chemotherapy, and radiation therapy are effective in eliminating or reducing primary tumor growth, their effectiveness for distant metastases is unfortunately limited. Traditional cancer treatments normally focus on reducing tumor burden in patients using external intervention. Stimulation of systemic self-defense against metastatic tumor has not been taken into much consideration. Accumulating evidences indicate that adaptive immunity generated by tumor therapy is correlated with good prognosis. Recently, new strategies to overcome tumor-associated immune suppression and to induce potent long-term anti-tumor immunity, are regarded as effective approaches of metastatic tumor treatment^2^.

Targeting host–tumor interaction has become an increasingly important and promising approach to tumor therapy. Myeloid derived suppressor cells (MDSCs) are found in majority of malignant tumors, including preclinical animal models and human patients^3^. MDSCs could inhibit the function of various types of immune cells mediating anti-tumor immunity, such as T cells, B cells, NK cells and dendritic cells, thereby play a pivotal role in tumor progression by suppressing both innate as well as adaptive immunity^5^,^6^,^7^,^8^. Given the high level of circulating MDSCs in patients with metastatic breast cancer^1^, it will be important to explore new anti-tumor immune therapies that target MDSCs.

In clinical studies, it has been shown that mild-to-moderate hyperthermia (38–45 °C) could work as a sensitizer for radiation therapy or chemotherapy^9^,^1^0. Mechanistically, hyperthermia at cytotoxic temperature (>43 °C) was found to be able to ablate tumor cells directly, resulting in the release of tumor antigen load and the induction of anti-tumor immunity^11^. In addition, hyperthermia could modulate the activities of immune cells, including APCs, T cells, and NK cells^11^,^12^,^13^. Interestingly, cryo-ablation of tumor can also modulate anti-tumor immunity^14^,^1^5. However, neither hyperthermia or cryosurgery alone has been proven to achieve long lasting effect to prevent tumor recurrence and metastasis so far.

To further improve the anti-tumor efficacy of thermal therapy, a novel therapeutic modality was developed for tumor cryo-thermal therapy by alternating liquid nitrogen (LN2) cooling and radio frequency (RF) heating of tumor tissues. Using the subcutaneous 4T1 murine mammary carcinoma model, it was shown that cryo-thermal therapy with only one cycle of rapid cooling followed by RF heating caused significant damage to tumor vessels, and marked tumor cell killing^16^. Moreover, we also found that the cryo-thermal therapy stimulated anti-tumor immune response, resulting in the increased infiltration of immunocytes, enhanced cytotoxicity T-lymphocyte (CTL), and elevated Th1 cytokines response^17^.

Studies have shown that the mode of tumor cell death upon thermal therapy has dramatic impact on the anti-tumor immune response^11^,^1^3,^18^,^19^. Necrosis is characterized by cellular breakdown, disruption of tissue architecture, release of the heat shock proteins (Hsps) associated with pro-inflammatory cytokine function^20^,^21^,^22^. Hsp70 plays an important role in anti-tumor immunity mediated by both innate and adaptive immune system^23^,^24^,^25^,^26^. Membrane bound Hsp70 triggers cytolysis attack by natural killer^27^. Extracellular Hsps released from necrotic tumor cells are regarded as potent adjuvants to facilitate the presentation of tumor antigens and the induction of anti-tumor immunity^28^,^2^9. Previous studies have shown that releasable Hsp70 could be detected 30 min after thermal therapy^30^. The Hsp-tumor peptide complex was phagocytized by APCs, such as dendritic cells (DC), though receptor-mediated endocytosis via several Hsp receptors^31^,^3^2. Hsp70 could provide the necessary “danger” signal that is required for DC activation and maturation, and induction of protective immunity^33^.

In this study, we investigated the release of Hsp70 after the local cryo-thermal therapy, and discovered a new function of Hsp70 in inducing MDSCs differentiation into mature DCs. Our findings provided important new insight into the mechanism of a robust systemic anti-tumor immune response after the cryo-thermal therapy.

## Results

### Therapeutic efficacy of cryo-thermal therapy

**Figure 1:**
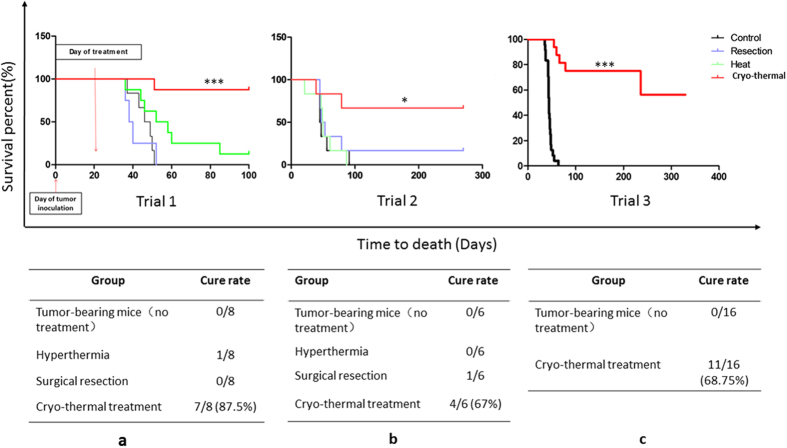
Survival curves comparing survival rate of mice in four groups (tumor-bearing control group; surgical resection group; hyperthermia group; cryo-thermal group). Cryo-thermal therapy improved long-term survival in comparison with surgical resection or RF hyperthermia therapy (*P < 0.05; ***P < 0.001). (**a**) Trial 1 comparing all 4 groups over a period of longer than 80 days (n = 8 per group; χ^2^ = 17.94, P < 0.0001). (**b**) Trial 2 comparing with all 4 groups over a period of longer than 250 days (n = 6 per group; χ^2^ = 4.73, P = 0.03). (**c**) Trial 3 comparing cryo-thermal and control groups over a period of some 350 days. (n = 16 per group;χ^2^ = 32.69, P < 0.0001).

To investigate the therapeutic effect of the cryo-thermal therapy, the long-term survival rate was analyzed in mice bearing 4T1 tumors. We have run three independent trials to compare the surgical resection, RF hyperthermia (50 °C for 15 min), and cryo-thermal therapy (pre-freezing at the temperature of −20 °C for 5 minutes followed by RF heating at the temperature of 50 °C for 10 minutes) on primary tumors on day 21 after tumor inoculation. In the first trial, all mice in the surgical resection and 7 of 8 mice in hyperthermia treated groups died by day 52 after tumor inoculation. In contrast, mice treated with the cryo-thermal therapy showed markedly increased survival. Except for one mouse died in 30 days after the treatment, the other 7 mice survived in good health condition in the following 3 months of observation (Fig. 1a). Similar results were obtained in the second trial (Fig. 1b). Because of the poor prognosis in RF hyperthermia and resection groups in the first two trials, we only investigated the therapeutic effect of cryo-thermal treatment in the third trial. 16 mice were treated on day 21 after tumor inoculation. As shown in Fig. 1c, during an observation period of ~350 days, all 16 mice in the tumor-bearing control group died; but in the cryo-thermal group, 11 of 16 (~69%) mice recovered and were in good health condition.

Moreover, during the observation period, tumor growth on the animal’s body surface was found in surgical resection (Supplementary Fig. S1a), and hyperthermia groups (Supplementary Fig. S1b), but none in the cryo-thermal group (Supplementary Fig. S2).

**Figure 2:**
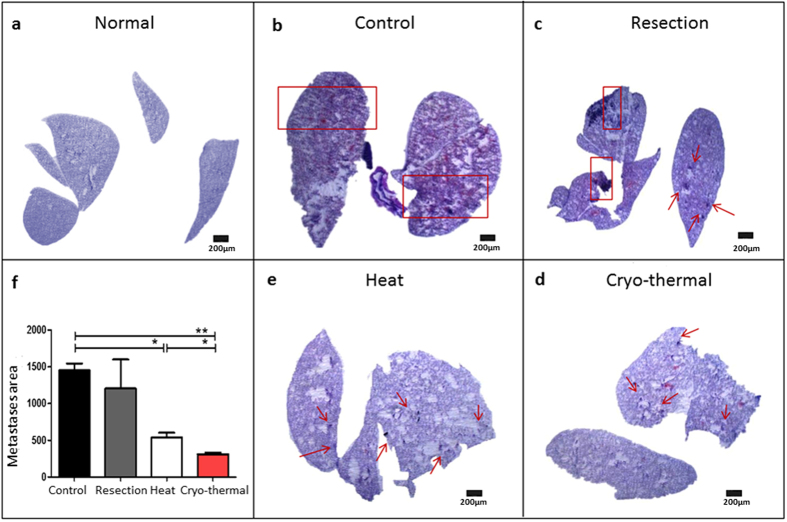
Cryo-thermal therapy significantly reduces lung metastases. (**a**) H&E staining of lung from normal mice. 5×, scale bar: 200 μm. (**b–e**) H&E staining of metastasis lesions in lungs on day 28 after treatment from four groups mice. (Red boxes and arrows indicate the metastasis lesions). 5×, scale bar: 200 μm. (**f**) Image Pro Plus software analyzed the metastasis areas of four groups (tumor-bearing control group; surgical resection group; RF hyperthermia group; cryo-thermal group). n = 4 per group.

Because metastatic tumor growth in the lungs is usually one of the main causes of death in 4T1 murine breast cancer model^34^, sections of the lung tissues from different groups were stained by H&E on day 28 after treatments (Fig. 2). It should be noted that since distal metastases were established before different treatments performed on day 21 after 4T1 tumor implantation, lung metastases were also shown in the cryo-thermal group, but exhibited substantial changes in 28 days after the treatment. As shown in Fig. 2b, mice in the control group developed tumor nodules in lungs. Tumor nodules were also found in lungs of the resection and hyperthermia groups (Fig. 2c,e). In contrast, less metastases were observed in the cryo-thermal group compared to other groups (Fig. 2d). In comparison, lesions in the control or the resection group invaded and damaged the pulmonary parenchyma, while lesions in the cryo-thermal or RF hyperthermia group were found smaller and non-invasive. Using Image Pro Plus software, the total cross-area of all lesions was quantified in each group. It was much smaller in the cryo-thermal group as compared with that of the control, resection and RF hyperthermia group, shown in Fig. 2f.

### Cryo-thermal therapy protected mice from tumor re-challenge

The observation described above clearly demonstrated that cryo-thermal therapy not only eradicated the primary tumor, but also markedly curbed the development of distant metastases, indicating a strong systemic anti-tumor immune response. To further confirm the induction of a systemic anti-tumor immunity, mice were re-challenged with 4T1 tumor cells on day 21 after tumor inoculation. The secondary tumor inoculation was performed on the opposite side with 1 × 10^5^ 4T1 tumor cells. As shown in Supplementary Fig. S3, the secondary tumor failed to grow in two of three mice from the cryo-thermal group. Furthermore, no surface metastases were found in the following 3 months. In comparison, all three mice in the control group developed the secondary tumors and died 22 days after tumor inoculation. In the RF hyperthermia group, the secondary tumor grew in two of the three mice, and both mice died in 22 days after tumor inoculation. The third mice developed distant metastases and died on day 30. These results further identified that the cryo-thermal therapy induced stronger systemic anti-tumor immunity.

### Reduction of immunosuppressive MDSCs and increase of splenic immune effector cells resulting from cryo-thermal therapy

**Figure 3:**
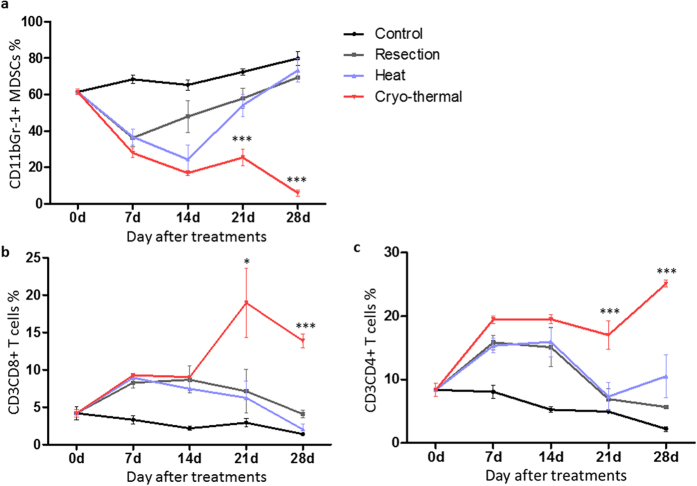
MDSCs and T cells dynamic changed (on day 0, 7, 14, 21 and 28 after treatment) in spleens of four groups (tumor-bearing control group; surgical resection group; hyperthermia group; cryo-thermal group). Four groups data were compared by one-way analysis of variance and every two groups data were compared by Bonferroni’s test (*P < 0.05; ***P < 0.001). (**a**) The percentages of MDSCs within spleen. MDSCs of the cryo-thermal group declined and significantly less than other goups on day 21 (R^2^ = 0.72, P < 0.0001) and 28 (R^2^ = 0.97, P < 0.0001). (**b**) The percentages of CD8^+^ T cells within spleen. T cells of the cryo-thermal group trend to increase and significantly more than other goups on day 21 (R^2^ = 0.43, P = 0.012) and 28 (R^2^ = 0.93, P < 0.0001). (**c**) The percentages of CD4^+^ T cells within spleen. T cells of the cryo-thermal group trend to increase and significantly more than other goups on day 21 (R^2^ = 0.58, P = 0.0008) and 28 (R^2^ = 0.91, P < 0.0001). n = 4 per group

The number of MDSCs inversely correlated with the CD3^+^ T cell frequency. MDSCs infiltration suppressed CD4^+^ and CD8^+^ T cells, thus contributing to the immune tolerance^10^. Previous studies showed tumor-infiltrating MDSCs were found in the 4T1 tumor model^35^. In addition, increased MDSCs were found in blood circulation, spleen, lung and liver, potentially contributing to the development of metastatic disease due to MDSCs-mediated immunosuppression^34^. We evaluated changes in the immunosuppressive and effector components of host immune system after the cryo-thermal therapy in the 4T1 tumor model. By flow cytometry, we analysed the dynamic changes of immune cells, including MDSCs, CD4^+^, and CD8^+^ T cells in spleen on day 7, 14, 21 and 28 after the treatment (Fig. 3). In the control group, the percentage of MDSCs increased continuously. On day 7, significant reductions of MDSCs in the spleen were observed in all therapeutic groups compared to the control group. Interestingly, from day 7 through day 28, in the resection and hyperthermia groups, MDSCs gradually increased to a level comparable to that in the control group. On the contrary, MDSCs continued to decline in the cryo-thermal group (Fig. 3a), which was accompanied by an increase of immune effector CD4^+^ and CD8^+^ T cells (Fig. 3b,c). On day 28, the percentage of CD4^+^ and CD8^+^ T cells was significantly higher in the cryo-thermal group than that in the resection and hyperthermia groups. CD4^+^ and CD8^+^ T cells in the resection and RF hyperthermia groups slightly increased right after the treatments, but started to gradually decline from day 14. On the other hand, CD4^+^ and CD8^+^ T cells in the control group maintained at the lowest level.

These results further demonstrated that cryo-thermal therapy was superior to the surgical resection in reducing MDSCs and increasing CD4^+^ and CD8^+^ T cells, which could contribute to the long term anti-tumor immune response and more favorable therapeutic outcomes-eradicating the primary tumors, protecting against tumor metastasis, and conferring resistance to tumor re-challenge.

### Increased intratumoral and serum levels of Hsp70 after cryo-thermal therapy

**Figure 4:**
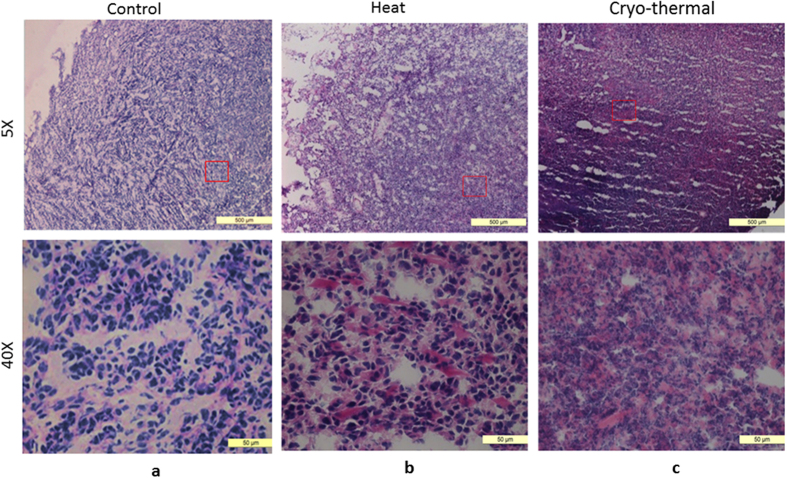
Histological analysis of tumor necrosis after different treatments by H&E staining. (**a**) Tumor-bearing control group; (**b**) Hyperthermia group; (**c**) cryo-thermal group. More pronounced necrosis was observed after cryo-thermal therapy, as compared to hyperthermia. 5×, scale bar: 500 μm (Highlight areas were selected by red boxes and 40× optical amplified below); 40×, scale bar: 50 μm.

Previous studies indicated that thermally induced anti-tumor immune response was dependent on the mode of cell death^11^,^1^3,^18^,^19^. Necrosis is characterized by cellular breakdown, and thought to be able to induce a more robust anti-tumor immune response. Therefore, we hypothesized that cryo-thermal therapy could augment tumor necrosis, leading to a more powerful anti-tumor immune response. We evaluated tumor necrosis after various treatments by H&E staining (Fig. 4). Indeed, more pronounced necrosis in the tumor periphery was observed after cryo-thermal therapy (Fig. 4c), as compared to other treatments (Fig. 4b).

Extracellular release of Hsps by necrotic cells is expected to contribute to tumor-specific immune response^28^,^2^9 and investigated in this study. The time point for measuring Hsp70 in tissue and interstitial fluids was decided according to observations of our other studies: i.e. M1 phenotype macrophage producing IFN-Y and other pro-inflammatory cytokines to create a pro-inflammatory microenvironment, which activates type-1 T cells, obviously increased in local tumor tissue in day 1 after treatment. So day 1 after treatment was the best time point to study the Hsp70 in local tumor tissue. In addition, the reason why day 3 after treatment was chosen to detect the Hsp70 in serum was due to DC maturation in day 5 after cryo-thermal treatment in our other study^36^, considering the time it takes for Hsp70 released from interstitial fluids into blood, and to stimulate DC maturation, day 3 after treatment was chosen as the time point for Hsp70 detection in serum.

**Figure 5:**
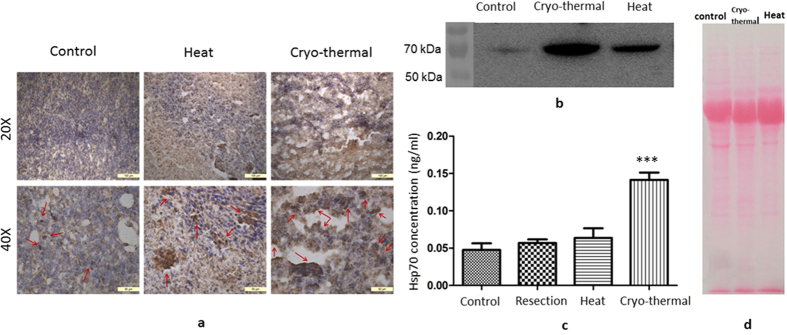
Hsp70 released from necrotic tumor cells in tumor stroma. (**a**) Histological analysis of Hsp70 in tumor stroma with different treatment by immunohistochemistry staining under microscopic observation (Brown areas represented released Hsp70 indicated by red arrows) 20×, scale bar: 500 um; 40×, scale bar: 50 um. (**b**) Western blot analysis of released Hsp70 in tumor interstitial fluid from different treatments (tumor-bearing control group; surgical resection group; hyperthermia group; cryo-thermal group) on day 3 after treatments. (**c**) ELISA detection of Hsp70 concentration in serum from all the four group mice on day 3 after treatment (n = 4 per group; R^2^ = 0.86, P = 0.0004). (**d**) Ponceaux staining of quantifying the same amount of proteins of 30 μg in all the samples. Local extracellular release of Hsp70 was much higher after cryo-thermal therapy as compared to that of the untreated control group, or hyperthermia group. The Hsp70 concentration in cryo-thermal group was significantly higher than other groups, n = 4 per group.

By immuno-histochemical staining we investigated Hsp70 release from necrotic cells in the tumor on day 1 after the treatments. As shown in Fig. 5a, only sparse brown signal of Hsp70 was detected in the untreated control group. Elevated Hsp70 was present in both the RF hyperthermia and the cryo-thermal groups, with more prominent tumor interstitial staining in latter. We further evaluated the extracellular release of Hsp70 by western blot analysis of tumor interstitial fluid (Fig. 5b). The result showed that local extracellular release of Hsp70 was much higher after the cryo-thermal therapy as compared to that of the untreated control group, or the RF hyperthermia group, consistent with the immunostaining data. In addition to the tumor local release of Hsp70, we also measured its serum level after the treatments. On day 3, serum samples were collected from the control, surgical resection, RF hyperthermia and cryo-thermal groups, and Hsp70 concentration was determined by ELISA. The result showed that serum Hsp70 was significantly higher after the cryo-thermal therapy with respect to other groups (Fig. 5c). Since Hsp70 can act as a danger signal and antigen-chaperone to activate both innate and adaptive immunity, the massive Hsp70 release from locally damaged tumor into circulation could broadly affect the host immunity system.

### Hsp70-dependent MDSCs differentiation into dendritic cells (DCs)

**Figure 6:**
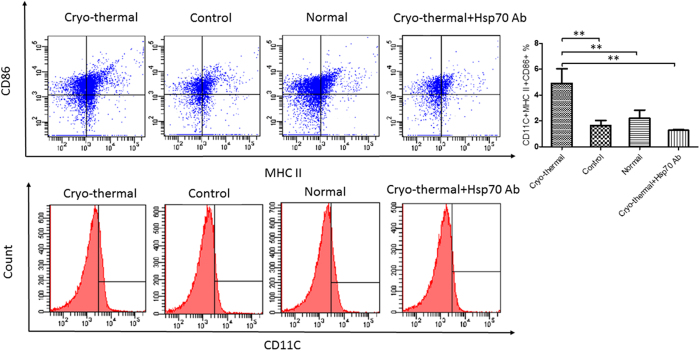
Hsp70 induced MDSC differentiate into matured DC *in vitro* study. DC (CD11C^+^) and mature DC (CD11^+^MHCII^+^CD86^+^) were analyzed by FASC assays, and results were performed in triplicates. The mature DCs was significantly elevated after culture with serum from the cryo-thermal treated mice in comparison to other groups. (**p < 0.01)

**Figure 7:**
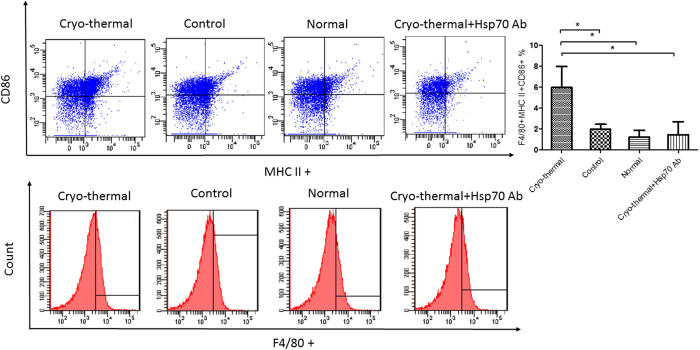
Hsp70 induced MDSC differentiate into matured macrophage *in vitro* study. Macrophage (F4/80^+^) and mature macrophage (F4/80^+^MHCII^+^CD86^+^) were analyzed by FASC assays, and results were performed in triplicates. The mature macrophage cells was significantly elevated after culture with serum from the cryo-thermal treated mice in comparison to other groups. (*p < 0.05).

MDSCs could terminally differentiate into DCs, granulocytes or macrophages under normal physiological condition. We explored the potential impact of Hsp70 on MDSC differentiation after the cryo-thermal therapy. On day 24 after tumor implantation, MDSCs from spleen were isolated using magnetic separation, and cultured for 24 hours with serum from healthy untreated mice, serum from tumor-bearing control mice, or serum collected on day 3 after cryo-thermal therapy. Cells were then stained for markers of mature DC (CD11c, MHC II, CD86) (Fig. 6), mature macrophage (F4/80, MHC II, CD86) (Fig. 7), and analyzed by flow cytometry. The expression of mature DC markers on MDSCs was significantly elevated after culture with serum from the cryo-thermal treated mice in comparison to serum from tumor-bearing control group or healthy mice. Importantly, the serum effect on the expression of mature DC markers was much diminished when an Hsp70 neutralizing antibody was added to the serum from the cryo-thermal treated mice (Fig. 6). Similar serum effect was observed for the expression of mature macrophage markers(Fig. 7).

**Figure 8:**
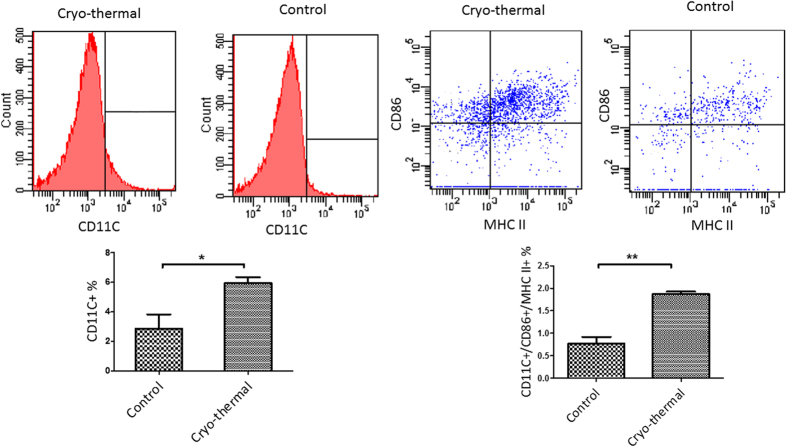
Hsp70 induced MDSC differentiation into mature DC in splenocytes on day 10 after cryo-thermal therapy *in vivo*. DC (CD11C^+^) and mature DC (CD11^+^MHCII^+^CD86^+^) were analyzed by FASC assays. The percentage of CD11c, CD86 and MHC II co-expression on MDSCs in cryo-thermal group was significantly higher than that of the tumor-bearing control group (n = 4 per group **P = 0.007).

**Figure 9:**
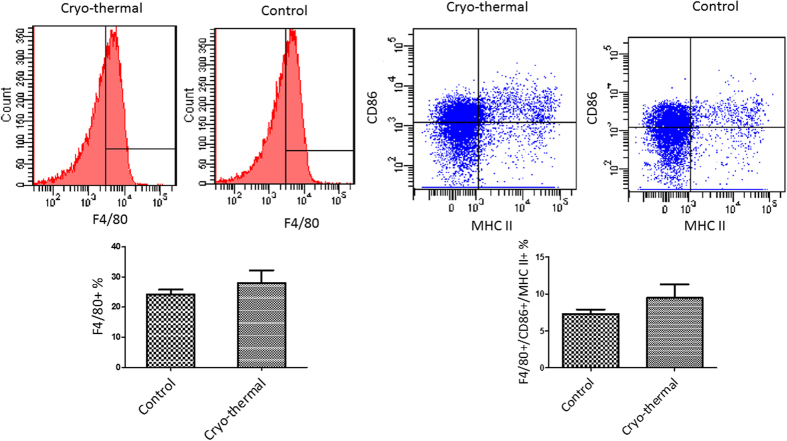
Hsp70 induced MDSC differentiation into mature macrophage in splenocyte on day 10 after cryo-thermal therapy *in vivo*. Macrophage (F4/80^+^) and mature macrophage (F4/80^+^MHCII^+^CD86^+^) were analyzed by FASC assays. There was a trend that the percentages of F4/80 expression and F4/80, CD86 and MHC II co-expression on MDSCs in the cryo-thermal group were higher compared to the tumor-bearing control group, but without statistical significance (n = 4 per group; P = 0.194).

To investigate the *in vivo* significance of the above-described *in vitro* findings, we compared splenic MDSCs isolated from tumor-bearing control mice and the cryo-thermal treated mice. The expression of mature DC markers (CD11c, MHC II, CD86) (Fig. 8), and mature macrophage markers (F4/80, MHC II, CD86) (Fig. 9) on MDSCs was evaluated by flow cytometry. The percentage of CD11c expression on MDSCs in the cryo-thermal group was much higher than the tumor-bearing control group. Moreover, the percentage of CD11c, CD86 and MHC II co-expression on MDSCs in cryo-thermal group was significantly higher than that of the tumor-bearing control group. On the other hand, there was a trend that the percentages of F4/80 expression and F4/80, CD86 and MHC II co-expression on MDSCs in the cryo-thermal group were higher compared to the tumor-bearing control group. These results were in complete agreement with our *in vitro* observations. Being one of the important immune stimulatory molecules, extracellular Hsp70 could facilitate presentation of tumor antigens, attract monocytes and neutrophils, and activate DCs. Results from the present study revealed a new function of Hsp70 in inducing MDSCs differentiation into mature DCs and macrophages, potentially contributing to the enhanced anti-tumor immune response after the cryo-thermal therapy.

### Reduced expression of arginase-1(Arg-1) and inducible nitric synthase(iNOS) in MDSCs after the cryo-thermal therapy

**Figure 10:**
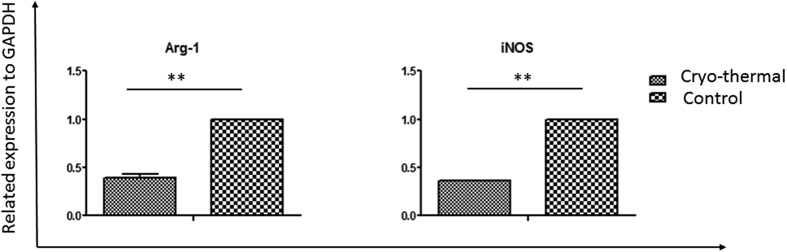
Detection of mRNA expression of Arg-1 and iNOS in MDSCs by RT-PCR. Cryo-thermal therapy significantly downregulated the expression of Arg-1 and iNOS in MDSCs. (**P < 0.01).

MDSCs affect anti-tumor immune responses by numerous mechanisms including strong activation of iNOS and Arg-1, leading to an intensive NO production and deprivation of amino acid L-arginine. To investigate functional impact of cryo-thermal therapy on MDSCs, we isolated MDSCs from spleen of treated mice and tumor-bearing control group, and the mRNA expression of arginase-1(Arg-1) and inducible nitric synthase (iNOS) in MDSCs was quantified by RT-PCR (Fig. 10). The results showed that cryo-thermal therapy significantly downregulated the expression of Arg-1 and iNOS in MDSCs.

## Discussion

In this work, a comprehensive study was performed to investigate the therapeutic effect of the cryo-thermal treatment in metastatic 4T1 murine mammary carcinoma tumor in comparison with surgical resection or RF hyperthermia treatment, and the possible mechanisms underlying the cryo-thermally stimulated immunological responses were investigated. The cryo-ablation treatment was not included as a reference because in our previous study^35^, the vascular damage induced by cryo-ablation under the given condition only was found limited, the same as the immune responses. The degree of apoptosis in cryo-ablation group was more significant than that in other groups (control, RF hyperthermia and cryo-thermal), and apoptotic death could induce immunosuppression^18^. It was also reported in other studies that, thermal ablation was mostly described to stimulate immune responses to certain degree, but cryo-ablation was observed to be both stimulatory and suppressive on the immune response depending on the degree of tissue necrosis and apoptosis. Thus, we excluded the cryo-ablation group in the present study which focused on immune response induced by cryo-thermal therapy. It was found that not only better therapeutic effect was achieved by the cryo-thermal therapy, but re-challenge of the 4T1 tumor was also inhibited, indicating a strong effective anti-tumor immunity response elicited. Further mechanistic studies showed significant reduction in immune suppressive cells (MDSCs) and increased immune effector cells (CD4^+^ and CD8^+^ T cells) accumulation in spleen after the cryo-thermal therapy. The resulted extensive tumor necrosis and elevated Hsp70 expression *in situ*, enabled releasing large amount of tumor antigens, which could bind to Hsp70 to relieve immune suppression and stimulate strong anti-tumor immune response. Moreover, up-regulated Hsp70 release in tumor interstitial fluid and serum was found to possibly relieve MDSC-mediated immunosuppression by differentiating MDSCs into mature dendritic cells for inducing protective anti-tumor immunity.

Tumor-induced immune suppression contributes to the limited efficacy of the current tumor therapy. It is likely that addressing the multiple immune deficits in cancer patients will be required for more effective therapy. MDSCs play a major role in the suppression of T cell activation and they sustain tumor growth, proliferation, and metastases. Regulation of MDSC recruitment, differentiation or expansion, and inhibition of the MDSC suppressive function will be useful in the control of tumor growth and progression. Optimization of approaches that simultaneously down regulate MDSC suppressor pathways, restore APC immune-stimulating activity, and expand tumor-reactive T cells will be useful in improving tumor therapy. Recently, aiming to manipulate the immunosuppressive cells in the tumor has become an emerging potential therapy strategy^37^. In this study, it was the most important that the cryo-thermal therapy significantly reduced immune suppression cells (MDSCs) accumulation. Immune suppression cells, such as MDSCs, TAMs and T-regs, are thought to inhibit the activities of tumor specific CD8^+^ T lymphocytes, and could be correlated with a poor prognosis in human cancer^38^,^39^,^40^. The cryo-thermal therapy decreased the immune suppression cells (MDSCs) greatly to relieve the immune suppression, and increased CD4^+^, CD8^+^ T lymphocytes to trigger specific strong anti-tumor immunity leading to long term survival of the treated mice. The great extent of tumor necrosis and damage-associated molecular pattern molecules (DAMPs) (e.g., ATP, calreticulin, Hsps and HMGB1) were induced and released from tumor necrotic cells providing both danger signals and antigenic peptides, which could act on the activation and maturation of DCs^41^,^4^2. It was generally accepted that DAMPs released during necrosis can lead to local pro-inflammatory and generate immune responses^43^,^4^4. Thus, necrotic antigen exposure in the“danger” signals might make the residual tumor cells more vulnerable to CTLmediated killing^34^.

We anticipated that the extent of tumor cells necrosis in tumor is deterministic to strong anti-tumor immune response and achieve therapeutic effect in the cryo-thermal therapy. The extent of necrosis is related to the thermal dose, changes of thermal dose might alter the degree of necrosis in the tumor and thus alter the immune response to tumor antigens. Extracellular Hsps derived from tumor cells released by cell necrosis are considered to act as danger signals^45^ and have been regarded as potent adjuvant facilitating presentation of tumor Ags and induction of antitumor immune response^46^,^47^,^48^. The specificity of the immunity generated was reported as being toward the antigenic peptides that were carried by the Hsps, rather than the Hsps itself^49^. Some studies found that hyperthermia could stimulate the innate immune and adaptive immune response, but the mechanism is not very clear. In general, hyperthermia may cause varying degrees of necrosis, induce intracellular Hsp release leading to NK activation, presenting the release of Hsp-antigen peptide to stimulate DC, and activation of T cells^50^. Though hyperthermia could generate large amounts of tumor debris as potential antigenic peptides complexes with Hsp70 for the induction of anti-tumor immunity^51^,^52^,^53^,^54^, the extent of tumor necrosis and released Hsp70 was found lower than the cryo-thermal therapy in our study.

To our knowledge, none of RF hyperthermia treatment was reported to stimulate long-lasting whole body anti-tumor immunity. But, several clinical trials have indicated that magnetic nanoparticle-mediated hyperthermia (MNHT) is a promising and novel approach to antitumor therapy^55^. Nanoparticle-mediated hyperthermia differs from RF heating alone given the introduction of magnetic nanoparticles (MNPs) that generate heat when exposed to an alternating magnetic field to cause tumor necrosis tumor and also induce HSP-peptide complex release to elicit systemic antitumor immune responses as reported^56^. Yanase *et al.* reported that hyperthermic system using magnetic nanoparticles produced HSP70-peptide via necrotic tumor cell death resulting in antitumor immunity, 50% of rats were protected from challenge with T-9 rat glioma cells^57^. In another study, Sato *et al.* identified that melanoma-targeted chemo-hyperthermia (using magnetite nanoparticles) therapy induced higher level of Hsp72 in the cell lysate resulting in stronger antitumor immune responses^58^.

It is also worthy to note that in the present study the animal tumor model with distal metastases was used, which allowed us to observe the effect on metastases and demonstrate the whole body anti-tumor immune response induced by local cryo-thermal treatment of primary tumors. Compared to other studies with RF heating, we used much lower thermal dose (shorter duration of 15 min, only one time treatment). Complete primary tumor cells necrosis and vascular damage was achieved through thermal and mechanical stress induced by an abrupt tissue temperature change and blood re-perfusion when heating imposed right after pre-freezing. Subsequently, significant Hsp70 release facilitated tumor antigen to stimulate DCs and trigger specific anti-tumor immunity in the whole body, which was not only observed in spleen but also proven by the resistance to tumor re-challenge. This is considered as a novel “green” approach and expected to have a great potential in future clinical applications.

More importantly, we found that released Hsp70 under thermal stress was able to enhance the differentiation of MDSCs into mature DCs, thus decreasing the number of MDSCs and reversed MDSCs mediated T cell suppression by reducing iNOS and arginase-1 expression of MDSCs as well as enhancing antitumor immune response. Large amount of released Hsp70 induced by the cryo-thermal therapy resulted in more MDSCs differentiation into mature DCs increasing antigen presentation and enhancing T effector cells activation to relieve the immune suppression and elicit stronger antitumor immune response. In the present study, thermally released Hsp70 triggered antitumor immune response as a critical immune stimulating molecule was investigated for the first time. Other released DAMPs resulted from tumor necrosis, such as ATP, calreticulin, and HMGB1 would be worthy further investigating.

In conclusion, the necrosis of primary tumor *in situ* after the cryo-thermal therapy induced up-regulated Hsp70 release leading to a relief of immune suppression and activation of the whole body strong anti-tumor immune response, which not only ablated primary tumor but also prevented metastatic progression, thus significantly enhanced the therapeutic effect.

## Methods

### Animal studies

All animal experiments were approved by the Animal Welfare Committee of Shanghai Jiao Tong University and experimental methods were performed in accordance with the guidelines of Shanghai Jiao Tong University Animal Care (approved by Shanghai Jiao Tong University Scientifique ethics committee). 4-week-old female BALB/mice were obtained from Shanghai SLAC Laboratory Animal Co., Ltd., China, then housed and fed sterile food with standard mice nutritional formula and sterile water in the isolated cages of 12-h light/dark cycle environment.

### Animal tumor model

The murine mammary carcinoma 4T1 cell line was provided by Shanghai First People’s Hospital, China. Cells were grown in DEME medium supplemented with 10% fetal bovine serum, plus 100 U/ml penicillin, and 100 g/ml streptomycin (Shanghai Sangon, China). 4T1 cells (1 × 10^5^) were injected subcutaneously into the right femoral region of BALB/mice. Tumor growth was monitored every other day thereafter. Tumor volume was estimated using the following formula: V (mm^3^) = π/6 × L (major axis) × W^2^ (minor axis).

### The thermal treatment procedures

The system developed in our laboratory^59^,^6^0 was composed of liquid nitrogen for cooling and radiofrequency (RF) for heating. A probe designed for the thermal treatment of subcutaneous mouse tumor was compatible with this system. To reduce the effect of contact thermal resistance and obtain a constant thermal delivery during the treatment, we custom-designed a probe with a concave-shaped tip of 10mm in diameter, suitable for the size of mouse tumors^60^. Three weeks after tumor inoculation when the average tumor size reached about 0.2 cm^3^, mice were divided into four groups: (1) tumor-bearing group without any treatment (control); (2) surgical resection group with primary tumor completely removed (resection); (3) hyperthermia group with radiofrequency (RF) heating on primary tumor at the temperature of 50 °C (controlled at the tumor bottom, Fig. S4 in supplementary) for 15 minutes (heat); (4) cryo-thermal group with freezing at the temperature of −20 °C for 5 minutes followed by RF heating at the temperature of 50 °C for 10 minutes on primary tumor (cryo-thermal). The mice were anesthetized with intra-peritoneal injection of 1.6% pentobarbital sodium (0.5 ml/100 g). The tumor site was sanitized with alcohol and iodine tincture before treatment. All the procedures were carried out aseptically.

### Long-term survival analysis and re-challenge with the 4T1 tumors

In three trials of experiments, the survival rate of a total of 88 mice was investigated. In addition, study of re-challenge with the 4T1 tumors was performed in mice after different treatments mentioned above (i.e. 3 mice used in each group). One hour after each treatment, approximately 1 × 10^5^ cells were transplanted subcutaneously into the opposite femoral region. The tumor growth at the primary and secondary sites was monitored.

### Isolation of splenic MDSC cells

Spleens were harvested from treated mice on day 3, and day 10. The splenocytes were separated by Gentle MACSD issociator (MiltnyiBiotec, Germany). Following the removal of RBCs and tissue debris, MDSC cells were isolated by using MDSC isolation micro-bread kit (MiltnyiBiotec, Germany) on MS columns. MDSCs with a purity of >90% were used for experiments.

### Flow cytometry analysis

The splenocytes from mice on day 0, 7,14, 21 and 28 after treatments were stained with FITC anti-mouse CD11b, PE anti-mouse Gr-1, FITC anti-mouse CD3, APC anti-mouse CD4, and APC anti-mouse CD8 for 30 minutes at 4 °C. MDSCs isolated from spleens of control group and the cryothermal group were incubated with AF488 anti-mouse CD11c, APC anti-mouse F4/80, Percp/cy5.5 anti-mouse CD86, and PE anti-mouse MHC II (Biolegend) for 30 minutes at 4 °C. Data were acquired on a FACSCalibur flow cytometer (BD Bioscience).

### Hematoxylin & Eosin (H&E) and immunohistochemistry stain

One day after after treatments, tumors were harvested and fixed in the isopentane at −80 °C. The frozen tissues were sliced into sections of 10 μm each at −20 °C. Some frozen sections were H&E stained. Another tumor frozen sections were fixed for 10 minutes in acetone at 4 °C, quenched by peroxidase, and then blocked for 30 minutes in 5% Bovine Serum Albumin (BSA). The tumor sections were incubated with anti-Hsp70 (Abcam, 1:1000) and biotinylated secondary antibody at 37 °C, 1 hour for each incubation. Tumor sections were further stained with streptavidin-HRP and diaminobenzidine (DAB) peroxidase, followed by counterstaining with hematoxylin for cell nuclei. All sections were examined microscopically.

On the day 28 after treatments, mouse lungs from each group were harvested and paraffin embedded. Paraffin-embedded tissues were sliced into sections of 7 μm each. The sections were stained with H&E. The metastatic lesions were evaluated by Image Pro Plus software in each groups.

### Hsp 70 ELISA

Serum Hsp70 in each group was determined by ELISA (ELISA kit, Abcam) on day 3 after treatments. Four biological replicates and two technical replicates were performed.

### Western blot analysis

Tumors were harvested one day after treatments. Tumor tissue was wrapped with Nylon filter paper with a pore size of 50 μm, and centrifuged at 2000 rpm in a 15 ml centrifuge tube for 20 minutes.Tumor interstitial fluid (TIF) was collected. 30 μg of total proteins were separated onto a 4–20% gradient Tris-Glycine precast gel and transferred to a PVDF membrane. Blot was probed with anti-Hsp70 (Abcam). Each shown Western blot was a representative from three separate experiments.

## MDSC differentiation assay

### *In vivo* study

Spleens were taken from mice on day10 after treatment. MDSCs were isolated as previously described. CD11c and F4/80 were used as DC cells and macrophage cells specific marker, respectively. CD86 and MHC II were used as the makers for mature DC and macrophage cells. All the markers were analyzed by flow cytometry.

### *In vitro* study

Mouse serum was harvested on day 3 after treatment. MDSCs (1 × 10^6^) were cultured in a 12-well culture plate for 24 hours at 37 °C in DMEM culture medium containing mouse serum from different treatment groups. After culturing for 24 hour, cells were analyzed by flow cytometry using various cell markers, including CD11c, F4/80, CD86 and MHC II. The effect of Hsp70 neutralizing antibody (Cell signaling technology) was assessed.

### Quantitative real-time PCR

Total RNA was isolated from MDSCs using TRIzol Reagent (Invitrogen, Carlsbad, CA, USA). First-strand cDNAs were synthesized using a PrimeScript RT reagent kit (TaKaRa). Quantitative real-time PCR was performed on ABI 7900HT sequence detection system, and SDS software (Applied Biosystems, Foster City, CA, USA) using SYBR Premix Ex Taq (TaKaRa). The primer sequences of mouse genes are: iNOS-forward ACATCGACCCGTCCACAGTAT, iNOS-reverseCAGAGGGGTAGGCTTGTCTC, Arg 1-forward TTGGGTGGATGCTCACACTG, Arg I-reverse GTACACGATGTCTTTGGCAGA, β-actin-forward CATGTACGTTGCTATCCAGGC, β-actin- reverse CTCCTTAATGTCACGCACGAT. The thermal-cycle condition was 95 °C for 30 s, 95 °C for 5 s, and 60 °C for 31 s, for 40 cycles. The mRNA level was calculated by the delta–delta Ct method using β-actin mRNA as the reference gene. All experiments were performed in biological triplicates.

### Statistical analysis

The GraphPad Prism software was used for all statistical analysis. Multiple groups data were compared by one-way analysis of variance and every two groups data were compared by Bonferroni’s test (*P < 0.05; **P < 0.01; ***P < 0.0001).

## Additional Information

**How to cite this article**: Zhu, J. *et al.* Cryo-thermal therapy elicits potent anti-tumor immunity by inducing extracellular Hsp70-dependent MDSC differentiation. *Sci. Rep.* **6**, 27136; doi: 10.1038/srep27136 (2016).

## Supplementary Material

Supplementary Information
