# Supplementary material for: Cryo-thermal therapy elicits potent anti-tumor immunity by inducing extracellular Hsp70-dependent MDSC differentiation
Source: Sci Rep. 2016 Jun 3;6:27136. doi: 10.1038/srep27136 (PMC4891716; doi:10.1038/srep27136)
Supplement: Supplementary Information [file srep27136-s1.pdf]

Cryo-thermal therapy elicits potent anti-tumor immunity by inducing extracellular Hsp70-dependent MDSC differentiation

Jun Zhu[1,2], Yan Zhang[1], Aili Zhang[1], Kun He[1], Ping Liu[1]*, Lisa X. Xu[1]*

[1]The School of Biomedical Engineering and Med-X Research Institute,

[2] Neurosurgery Department, Ruijin Hospital,School of Medicine,

Shanghai Jiao Tong University, Shanghai, China

Corresponding Author (*):

Lisa X. Xu: lisaxu@sjtu.edu.cn

Ping Liu: pingliu@sjtu.edu.cn

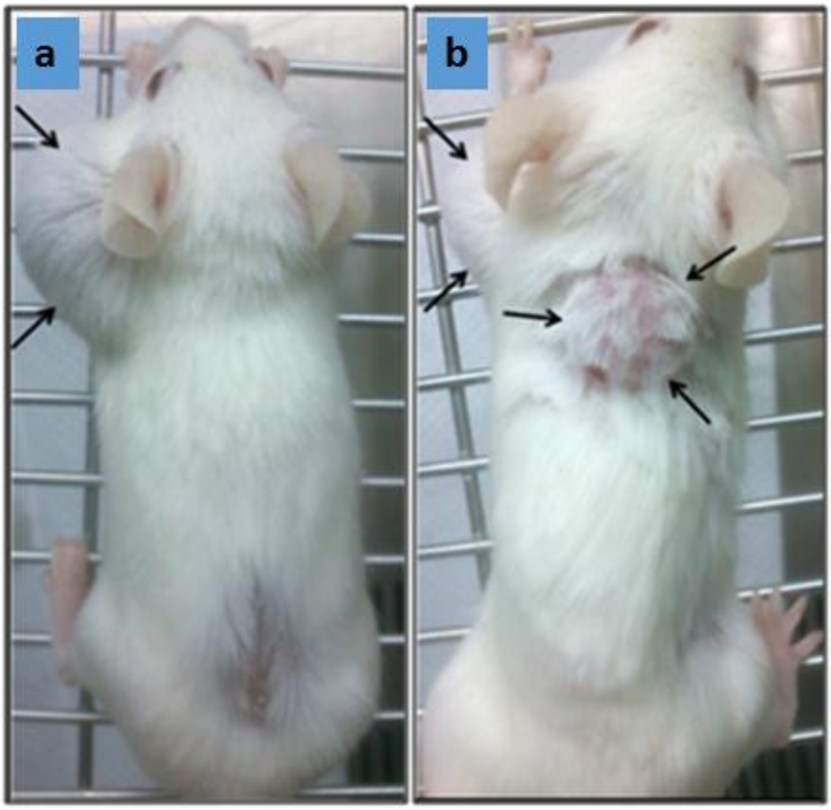

Supplementary figure 1 Surface metastasis after three weeks treatment was found in (A) Surgical resection group and (B) hyperthermia group.

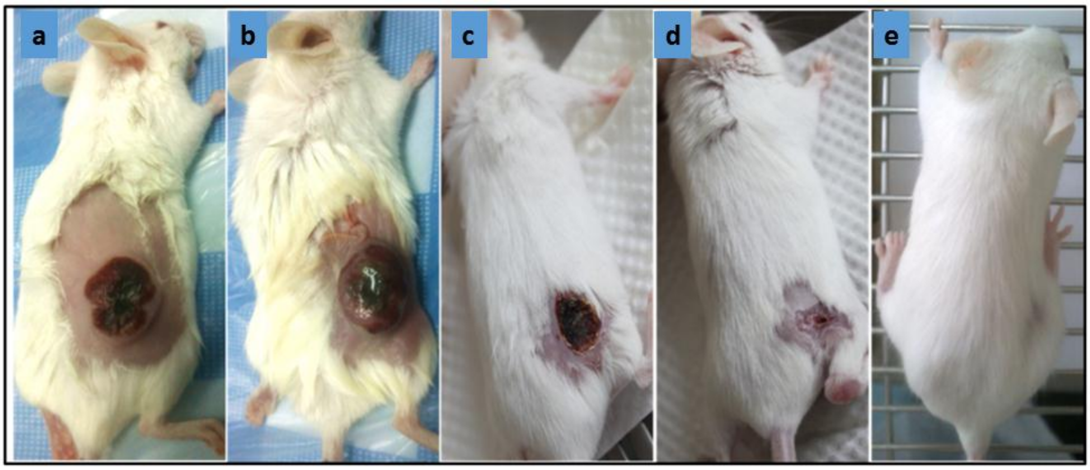

Supplementary figure 2 Tumor in situ changes after recieving cryo-thermal therapy in the survival mice without surface metastasis. (A) Before treatment; (B) After treatment; (C) One week after treatment ; (D) Three weeks after treatment; (E) Six weeks after treatment.

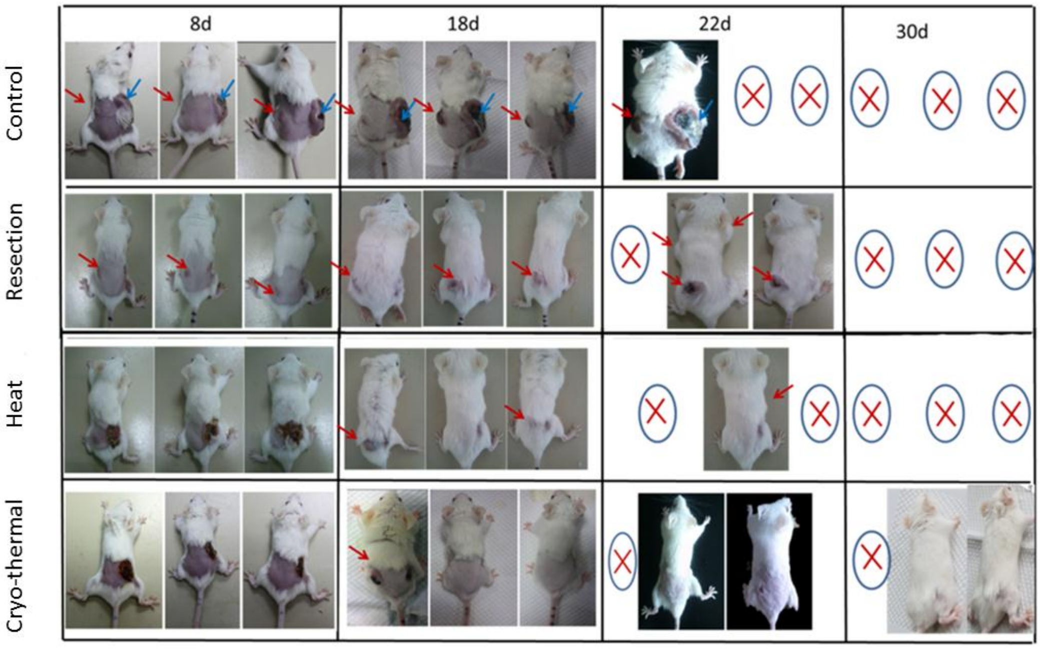

Supplementary figure 3 Re-challenge with the 4T1 tumor cells was carried out in the tumor-bearing control and each treated group. The anti-tumor immunity induced by cryo-thermal therapy potentially inhibited the local tumor recurrence and protected mice from re-challenge with the 4T1 tumor cells. (Blue arrows indicated the primary tumors. Red arrows indicated the second innoculated tumors; Blue oval circles with red crosses meant mouse was dead.) n=3 per group

The RF equipment specifications

The RF system used was developed in our lab. The frequency of the RF was 460 *kHZ*, and a temperature control mode was used when the temperature of the bottom edge of the tumor was controlled to be at 50℃. The control strategy was used for all the animals. We supplied a simulative temperature distribution during RF heating with this temperature control mode **(Supplementary figure** 4). The experimentally recorded RF emission voltage on the surface of the electrode was used as input to the model. And both the electrical field and temperature information was obtained. Results showed that with this heating mode, all the tumor region could be controlled at a temperature higher than 50℃ and the highest temperature was a little more than 60℃. The temperature inside the tumor was not uniform during the heating. The closer the tissue was to the electrode, the higher the temperature. According to the simulated temperature shown below, the highest temperature inside the whole tumor was around 60℃,

which was much lower than the temperature used in other RF ablation studies. Although we did not monitor the temperature of the animal's rectum during our study, all the experimental mice survived and appeared normally after treatment.

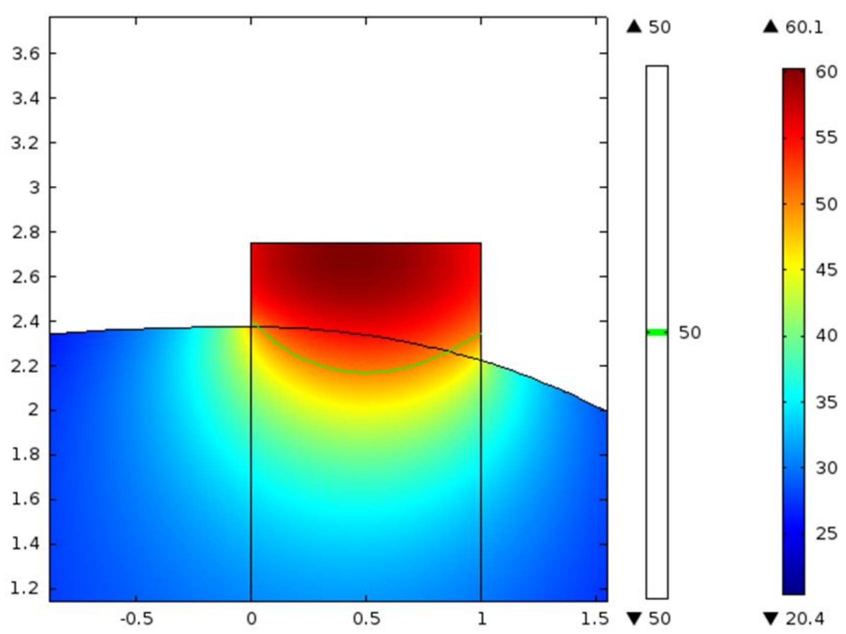

Supplementary figure 4 **The simulated temperature distribution during the RF heating process.** The input was the recorded voltage on the electrode surface. The green color indicated the 50°C thermal line monitored throughout the treatment. It was almost at the edge of the tumor as the depth of the tumor used in our experiment was around 5-6mm. The results showed that highest temperature inside the tumor was a little higher above 60°C.

	Control	Resection	Heat	Cryo-thermal
**Weight (g) (mean±SD)**				
**Trail 1**	25.33±2. 09	26.24±1. 22	25.33±1. 39	24.85±1. 10
**Trail 2**	24.20±0. 56	24.97±0. 68	24.35±1. 37	24.13±0. 85
**Trail 3**	23.92±0. 57			23.32±1. 17
**Volume (cm^3) (mean±SD)**				
**Trail 1**	0.27±0. 10	0.28±0. 08	0.26±0. 03	0.25±0. 06
**Trail 2**	0.29±0. 07	0.23±0. 03	0.27±0. 07	0.22±0. 01
**Trail 3**	0.26±0. 06			0.24±0. 05

Supplementary Table 1 The weight and tumor volume of mice used in each group prior to the treatment of the three trials.
